# ML-171 Attenuates Pentylenetetrazole-Associated Oxidative and Apoptotic Injury Without Robust Suppression of Seizure Expression: An In Vitro and In Vivo Study

**DOI:** 10.3390/ijms27146269

**Published:** 2026-07-14

**Authors:** Ahmet Ozan Kaleci, Ahmet Altun, Ahmet Şevki Taşkıran, Mustafa Özkaraca, İhsan Bağçivan

**Affiliations:** 1 Department of Medical Pharmacology, Faculty of Medicine, Sivas Cumhuriyet University, 58140 Sivas, Turkey; md.ahmetaltun@gmail.com (A.A.); ibagcivan@cumhuriyet.edu.tr (İ.B.); 2 Department of Physiology, Faculty of Medicine, Sivas Cumhuriyet University, 58140 Sivas, Turkey; ahmettaskiran@cumhuriyet.edu.tr; 3 Department of Veterinary Pathology, Faculty of Veterinary Medicine, Sivas Cumhuriyet University, 58140 Sivas, Turkey; mustafaozkaraca@cumhuriyet.edu.tr

**Keywords:** epilepsy, pentylenetetrazole, NOX-1, ML-171, NADPH Oxidase, oxidative stress, apoptosis, SH-SY5Y cells, hippocampus, kindling

## Abstract

Oxidative stress and apoptosis contribute to seizure-associated neuronal injury, but the effects of ML-171, a pharmacological NOX-1 inhibitor, on PTZ-associated oxidative and apoptotic injury remain insufficiently defined. This study evaluated the effects of the NOX-1 inhibitor ML-171 in pentylenetetrazole (PTZ)-induced cellular and animal models of neuronal injury. SH-SY5Y cells were exposed to PTZ (30 mM, 24 h) after pretreatment with ML-171 or valproic acid (VPA), and cell viability, redox status, apoptosis-related proteins, and Annexin V-based cell death were assessed. Adult male Wistar Albino rats were subjected to PTZ kindling and acutely treated with VPA or ML-171 (0.1, 1, or 10 mg/kg); behavioral seizures, electrocorticographic activity, hippocampal oxidative stress, apoptotic markers, histopathology, and NOX-1 immunoreactivity were evaluated. ML-171 attenuated PTZ-induced cellular injury in SH-SY5Y cells within a restricted concentration window and reduced oxidant burden, oxidative stress index, and apoptotic signaling. In PTZ-kindled rats, ML-171 improved hippocampal oxidative and apoptotic markers but did not robustly suppress behavioral or electrophysiological seizure parameters. Histopathological injury and NOX-1 immunoreactivity showed region- and dose-dependent responses. These findings suggest that ML-171 primarily attenuates PTZ-associated oxidative and apoptotic injury rather than exerting a conventional antiseizure effect.

## 1. Introduction

Epilepsy is a chronic neurological disorder characterized by a predisposition to spontaneous and recurrent seizures, and it is a significant public health problem, estimated to affect more than 50 million people worldwide [1]. Despite the expansion of current antiseizure drug options, seizure control is not adequately achieved in 20–30% of patients, and drug resistance is observed [2]. This situation maintains the need for new therapeutic targets that focus not only on seizure suppression but also on the mechanisms that determine seizure-related tissue damage [3].

The ionic imbalance resulting from increased metabolic rate and overstimulation of neurons during seizure activity leads to an increase in the production of reactive oxygen species [4]. Oxidative stress occurs when the balance between oxidant production and antioxidant defense capacity shifts towards oxidant production. This increase in oxidative stress causes molecular damage in cells [5]. Brain tissue is more vulnerable to oxidative damage due to its high oxygen consumption, polyunsaturated fatty acid-rich membrane structure, and relatively limited antioxidant capacity [6]. Therefore, oxidative stress accompanying seizures is not merely a biochemical consequence of neuronal hyperactivity but a major contributor to seizure-associated neuronal injury [7].

Neuronal hyperactivity during epileptic seizures mediates oxidative stress, disrupting the balance between proapoptotic and antiapoptotic proteins and consequently causing apoptosis in neurons through caspase activation [8,9]. In this context, changes in the Bax/Bcl-2 balance and caspase-3 activation are frequently used markers for monitoring seizure-related neuronal damage at the molecular level [10].

Attempting to suppress the increased oxidative stress during seizures with only exogenous antioxidants is relatively limited [11]. Therefore, identifying enzymatic sources of reactive oxygen species and targeting these sources pharmacologically represents a rational strategy for limiting ROS generation upstream rather than relying solely on downstream antioxidant scavenging [12]. In this context, the therapeutic targeting of NADPH oxidase (NOX) enzymes is noteworthy due to their vital roles in the production of reactive oxygen species [13].

The NOX enzyme family consists of transmembrane oxidoreductase systems that generate superoxide and related reactive oxygen species by transferring electrons from NADPH to molecular oxygen [14]. Several NOX isoforms have been implicated in neurological disorders, but their cellular distribution and pathological roles are not identical. NOX2 is frequently associated with microglial activation and neuroinflammatory oxidative injury, whereas NOX4 has been linked to sustained redox signaling, vascular dysfunction, and tissue remodeling in different neurological and neurodegenerative contexts [15,16]. These observations indicate that NOX-derived oxidative stress is not mediated by a single uniform enzymatic source, but rather reflects isoform-, cell type-, and disease context-dependent mechanisms.

The rationale for focusing on NOX-1 in the present study was based on its reported expression in neuronal structures and its association with oxidative injury in experimental neurological disease models [16,17]. Unlike broader antioxidant approaches, targeting NOX-1 may provide an upstream strategy for modulating enzymatic ROS generation in neuronal injury conditions. Because seizure activity is accompanied by excessive oxidant production and apoptosis-related neuronal damage, NOX-1-associated redox signaling may contribute to the secondary injury cascade triggered by PTZ exposure. Therefore, evaluating ML-171 allowed us to examine whether pharmacological NOX-1-targeted modulation could attenuate PTZ-associated oxidative and apoptotic injury while also determining whether these effects were accompanied by changes in seizure expression.

ML-171 is a compound previously identified as a NOX-1 inhibitor in earlier studies and has been shown to suppress the NOX-1-related oxidative response in cellular systems [18]. Although data exist regarding the neuroprotective effects of approaches targeting NOX inhibition, the effects of pharmacological NOX-1-targeted modulation in the context of epileptic seizures, particularly its role in acute seizure-associated injury processes, have not been sufficiently clarified [19,20,21].

Pentylenetetrazole (PTZ) is a chemical convulsant that can induce seizures by disrupting GABA (A)-mediated inhibition and is widely used in epilepsy research [22,23]. The kindling protocol, created by repeating subconvulsive PTZ applications, allows for modeling the establishment of epileptic susceptibility; while evaluating the acute PTZ-triggered seizure response in fully kindled animals provides a suitable experimental framework for examining both seizure phenotype and seizure-related acute tissue damage [24]. In addition, the PTZ-induced SH-SY5Y neuronal cellular damage model allows for the assessment of oxidative stress, cell viability, and apoptosis responses at the cellular level [25]. Therefore, the present study tested whether pharmacological NOX-1-targeted modulation with ML-171 could attenuate PTZ-induced oxidative stress, apoptosis-related signaling, and neuronal injury, and whether these injury-related effects were accompanied by changes in behavioral and electrophysiological seizure expression.

## 2. Results

### 2.1. In Vitro Studies

#### 2.1.1. ML-171 Attenuated PTZ-Induced Cytotoxicity in SH-SY5Y Cells

PTZ exposure markedly reduced SH-SY5Y cell viability compared with the untreated control group (*p* < 0.0001), confirming the establishment of an in vitro PTZ-induced cytotoxicity model. ML-171 pretreatment significantly attenuated this reduction at 80, 40, 20, and 10 µM compared with PTZ alone (*p* < 0.001, *p* < 0.0001, *p* < 0.0001, and *p* < 0.0001, respectively), whereas 160 µM ML-171 did not produce a significant protective effect (*p* > 0.05). Among the effective concentrations, the most pronounced recovery was observed at 40 and 20 µM.

**Figure 1:**
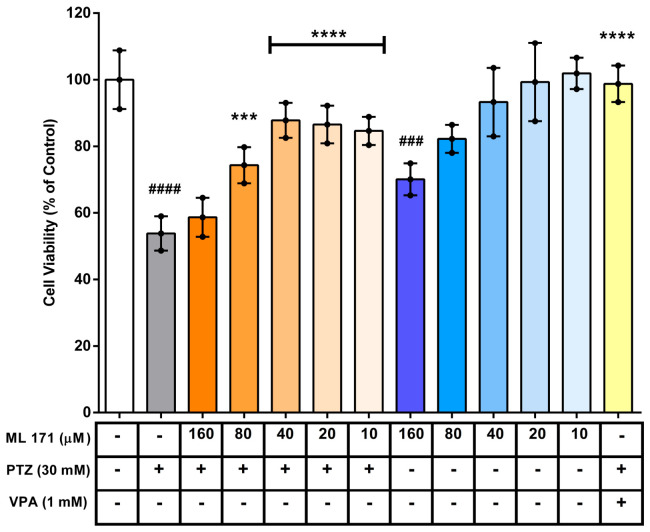
Effects of ML-171 on cell viability after PTZ-induced cytotoxicity in SH-SY5Y cells. SH-SY5Y cells were pretreated with ML-171 (10, 20, 40, 80, or 160 µM) or VPA (1 mM) for 3 h, followed by exposure to PTZ (30 mM) for 24 h. Cell viability was assessed using the XTT assay and expressed as a percentage of the untreated control group. Data are presented as mean ± SD from three independent experiments, each with three technical replicates; replicate values were averaged within each experiment, and the experiment mean was used as the statistical unit. Data were analyzed by one-way ANOVA followed by Tukey’s multiple-comparisons test. Different column colors distinguish the experimental groups and concentrations. ### *p* < 0.001 and #### *p* < 0.0001 vs. the untreated control group; *** *p* < 0.001, and **** *p* < 0.0001 vs. the PTZ group. PTZ, pentylenetetrazole; VPA, valproic acid.

When administered alone, ML-171 showed a concentration-dependent tolerability profile. ML-171 at 160 µM significantly reduced cell viability compared with the control group (*p* < 0.001), whereas 80, 40, 20, and 10 µM did not significantly affect cell viability (*p* > 0.05). VPA, used as a reference treatment, significantly reversed PTZ-induced loss of viability (*p* < 0.0001 vs. PTZ). Although 80 µM ML-171 attenuated PTZ-induced cytotoxicity, 40 µM was selected for subsequent in vitro experiments as a robustly protective concentration with a clearer tolerability margin when administered alone (Figure 1).

#### 2.1.2. ML-171 Reduced PTZ-Induced Oxidant Burden Without Significantly Restoring TAS in SH-SY5Y Cells

PTZ exposure significantly decreased TAS levels compared with the untreated control group (*p* < 0.05). Pretreatment with 40 µM ML-171 or VPA did not significantly increase TAS levels compared with the PTZ group (*p* > 0.05).

**Figure 2:**
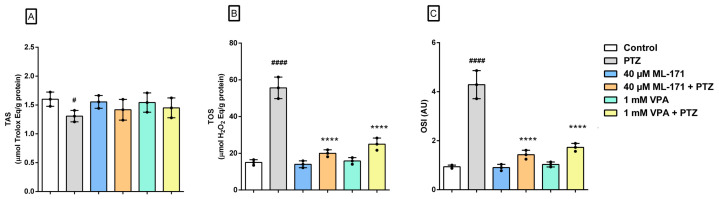
Effects of ML-171 on oxidative stress parameters after PTZ-induced injury in SH-SY5Y cells. SH-SY5Y cells were pretreated with ML-171 (40 µM) or VPA (1 mM) for 3 h and then exposed to PTZ (30 mM) for 24 h. Oxidative stress was assessed by measuring (**A**) TAS, (**B**) TOS, and (**C**) OSI levels. Data are presented as mean ± SD from three independent experiments, each with two technical replicates; replicate values were averaged within each experiment, and the experiment mean was used as the statistical unit. Data were analyzed by one-way ANOVA followed by Tukey’s multiple-comparisons test. # *p* < 0.05 and #### *p* < 0.0001 vs. the control group; **** *p* < 0.0001 vs. the PTZ group. PTZ, pentylenetetrazole; VPA, valproic acid; TAS, total antioxidant status; TOS, total oxidant status; OSI, oxidative stress index.

In contrast, PTZ markedly increased both TOS and OSI compared with control cells (*p* < 0.0001 for both parameters), indicating a robust oxidative stress response. Pretreatment with 40 µM ML-171 significantly reduced PTZ-induced elevations in TOS and OSI (*p* < 0.0001 for both comparisons). A similar reduction was observed in the VPA + PTZ group (*p* < 0.0001 for both parameters). ML-171 alone and VPA alone did not significantly alter TAS, TOS, or OSI compared with the control group (*p* > 0.05). These results show that ML-171 pretreatment reduced PTZ-induced increases in TOS and OSI, whereas its effect on TAS did not reach statistical significance (Figure 2).

#### 2.1.3. ML-171 Attenuated PTZ-Induced Apoptosis-Related Signaling in SH-SY5Y Cells

PTZ exposure significantly increased Bax (*p* < 0.0001) and cleaved caspase-3 (*p* < 0.0001) levels and decreased Bcl-2 levels (*p* < 0.0001) compared with the untreated control group, indicating a shift toward a pro-apoptotic profile. Pretreatment with 40 µM ML-171 significantly reduced Bax (*p* < 0.001) and cleaved caspase-3 (*p* < 0.0001) levels and increased Bcl-2 levels (*p* < 0.001) compared with PTZ-treated cells. VPA produced a similar pattern, reducing Bax (*p* < 0.001) and cleaved caspase-3 (*p* < 0.0001) levels and increasing Bcl-2 levels (*p* < 0.0001) compared with the PTZ group.

Consistent with these changes, the Bax/Bcl-2 ratio was significantly increased following PTZ exposure compared with control cells (*p* < 0.0001). Pretreatment with either 40 µM ML-171 or VPA significantly reduced the PTZ-induced increase in the Bax/Bcl-2 ratio (*p* < 0.0001 for both comparisons). ML-171 alone and VPA alone did not significantly alter Bax, Bcl-2, cleaved caspase-3 levels, or the Bax/Bcl-2 ratio compared with the control group (*p* > 0.05 for all comparisons).

**Figure 3:**
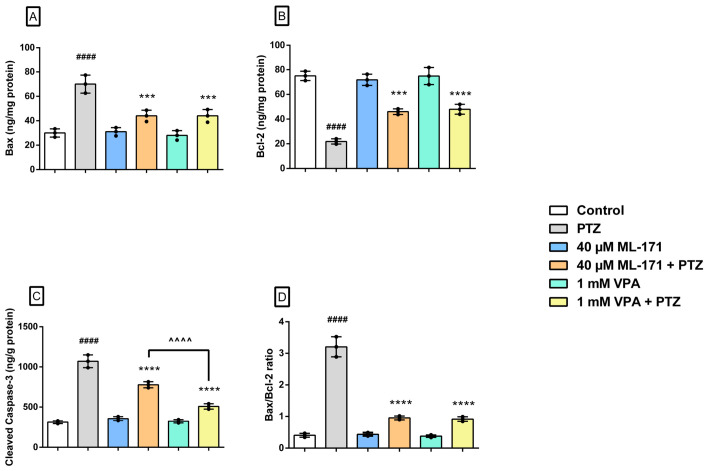
Effects of ML-171 on apoptotic markers in PTZ-exposed SH-SY5Y cells. SH-SY5Y cells were pretreated with ML-171 (40 µM) or VPA (1 mM) for 3 h before exposure to PTZ (30 mM) for 24 h. (**A**) Bax, (**B**) Bcl-2, and (**C**) cleaved caspase-3 levels were measured by ELISA. (**D**) The Bax/Bcl-2 ratio was calculated from the corresponding Bax and Bcl-2 values. Data are presented as mean ± SD from three independent experiments, each with two technical replicates; replicate values were averaged within each experiment, and the experiment mean was used as the statistical unit. Individual points represent the means of independent experiments. Data were analyzed by one-way ANOVA followed by Tukey’s multiple-comparisons test. #### *p* < 0.0001 vs. the control group; *** *p* < 0.001 and **** *p* < 0.0001 vs. the PTZ group; ^^^^ *p* < 0.0001 for the indicated comparison between treatment groups. PTZ, pentylenetetrazole; VPA, valproic acid; Bax, Bcl-2-associated X protein; Bcl-2, B-cell lymphoma 2.

Overall, these results indicate that ML-171 pretreatment attenuated PTZ-induced pro-apoptotic alterations in SH-SY5Y cells, with a pattern broadly similar to that observed in the VPA-treated reference group (Figure 3).

#### 2.1.4. ML-171 and Valproic Acid Attenuated PTZ-Induced Alterations in Viable and Apoptotic Cell Populations

**Figure 4:**
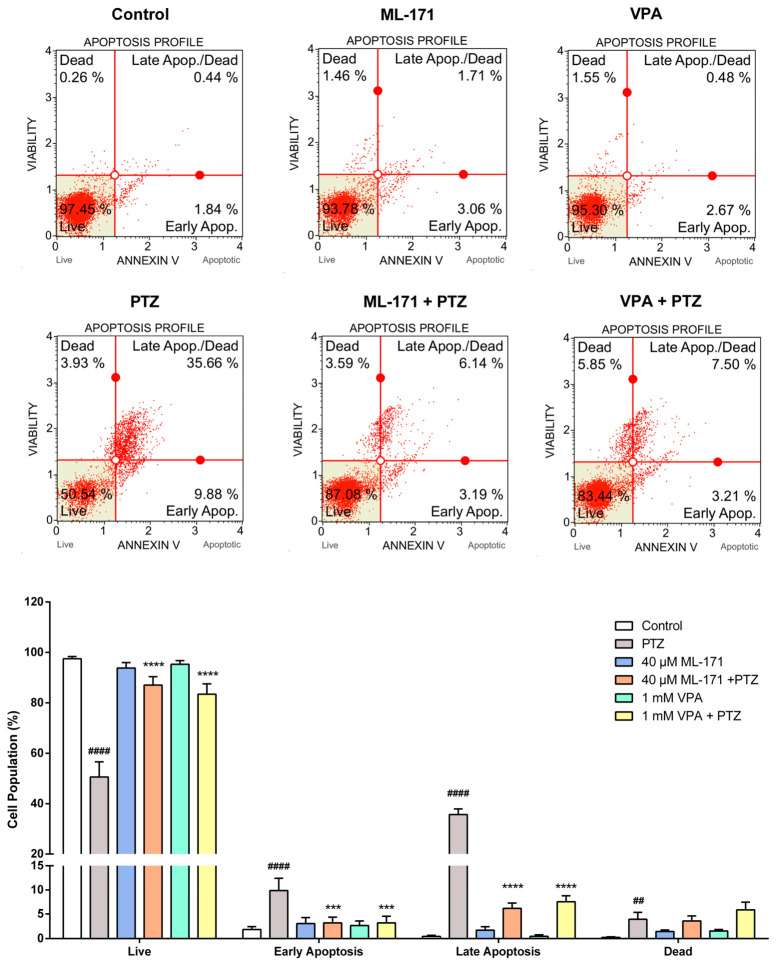
Effects of ML-171 pretreatment on apoptotic cell death after PTZ-induced injury in SH-SY5Y cells. Representative flow cytometry plots from one independent experiment are shown in the upper panels for the control, ML-171, VPA, PTZ, ML-171 + PTZ, and VPA + PTZ groups. In the representative flow-cytometry plots, red dots represent individual cellular events, and red lines indicate the predefined quadrant boundaries. Cells were pretreated with ML-171 or VPA for 3 h before PTZ exposure, and quantitative analysis of viable, dead, early apoptotic, and late apoptotic cell populations is shown in the lower panel. Data are presented as mean ± SD from four independent experiments and were analyzed by one-way ANOVA followed by Tukey’s multiple-comparisons test. Technical replicates, if present, were not treated as independent observations. ## *p* < 0.01 and #### *p* < 0.0001 vs. the control group; *** *p* < 0.001 and **** *p* < 0.0001 vs. the PTZ group. Percentages were derived from the predefined Muse Annexin V/Dead Cell quadrants. PTZ, pentylenetetrazole; VPA, valproic acid.

SH-SY5Y cells were pretreated with ML-171 or VPA for 3 h before PTZ exposure. PTZ markedly shifted the cell-death profile, producing a significant reduction in the viable-cell population (*p* < 0.0001) together with significant increases in early apoptosis (*p* < 0.0001), late apoptosis (*p* < 0.0001), and the dead-cell fraction (*p* < 0.01) compared with the control group. ML-171 pretreatment significantly restored the viable-cell population and reduced both early and late apoptotic fractions compared with PTZ alone (*p* < 0.0001 for all three comparisons), whereas the dead-cell fraction was not significantly altered (*p* > 0.05). VPA + PTZ showed a broadly similar protective pattern, with recovery of the viable-cell population and reductions in early and late apoptotic fractions (*p* < 0.0001 for all three comparisons); however, the dead-cell fraction was not significantly reduced compared with PTZ alone (*p* > 0.05). ML-171 or VPA alone did not induce a pronounced apoptotic shift compared with control cells (*p* > 0.05 for all comparisons) (Figure 4).

### 2.2. In Vivo Studies

#### 2.2.1. ML-171 Showed Limited Effects on Behavioral and Electrophysiological Seizure Expression in PTZ-Kindled Rats

Repeated PTZ administration induced severe seizure activity in rats, as reflected by high modified Racine scores and frequent epileptiform spike activity. Valproic acid significantly reduced the modified Racine score compared with the PTZ group (*p* < 0.01) and significantly prolonged first myoclonic jerk latency (*p* < 0.001). In addition, valproic acid significantly decreased epileptiform spike number compared with PTZ alone (*p* < 0.01).

**Figure 5:**
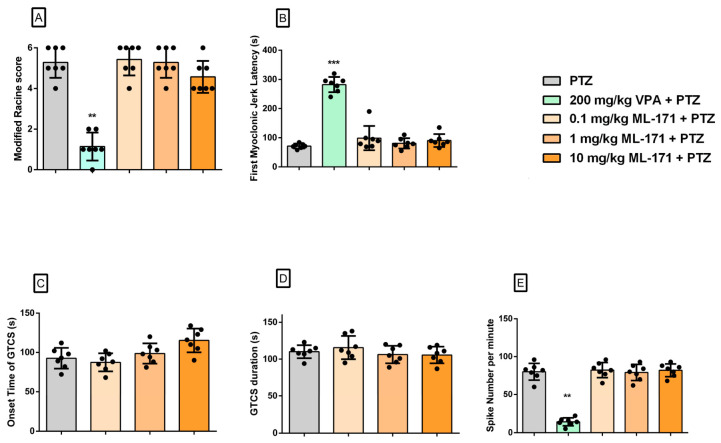
Effects of ML-171 on behavioral and electrophysiological seizure parameters in PTZ-kindled rats. (**A**) Modified Racine score, (**B**) first myoclonic jerk latency, (**C**) onset time of generalized tonic–clonic seizures (GTCS), (**D**) GTCS duration, and (**E**) epileptiform spike number per minute. GTCS duration was analyzed only in animals that developed GTCS; animals without GTCS during the 30-min recording period were treated at the 1800-s observation limit for the onset-latency analysis, as detailed in the Methods. Panel A is presented as median [IQR], and panels B–E as mean ± SD; individual points represent separate animals. Modified Racine scores were analyzed using the Kruskal–Wallis test followed by Dunn’s multiple-comparisons test. ** *p* < 0.01 and *** *p* < 0.001 vs. the PTZ group. PTZ, pentylenetetrazole; VPA, valproic acid; GTCS, generalized tonic–clonic seizure; IQR, interquartile range.

In contrast, ML-171 did not significantly alter the modified Racine score, first myoclonic jerk latency, or epileptiform spike number at any tested dose compared with the PTZ group (*p* > 0.05 for all comparisons). Although 10 mg/kg ML-171 tended to prolong the onset time of generalized tonic–clonic seizures, this effect did not reach statistical significance after multiple-comparison correction (*p* > 0.05). Similarly, GTCS duration did not differ significantly among the PTZ and ML-171-treated groups (*p* > 0.05). Overall, these findings indicate that ML-171 did not exert robust antiseizure activity under the present experimental conditions and clearly differed from VPA in its behavioral and electrophysiological profile (Figure 5).

#### 2.2.2. ML-171 Reduced Hippocampal Oxidant Burden in PTZ-Kindled Rats

PTZ administration significantly disrupted hippocampal oxidative balance, as demonstrated by a decrease in total antioxidant status (TAS; *p* < 0.01 vs. control) and marked increases in total oxidant status (TOS; *p* < 0.001 vs. control) and oxidative stress index (OSI; *p* < 0.001 vs. control). Valproic acid did not significantly increase TAS compared with the PTZ group (*p* > 0.05), but significantly reduced both TOS and OSI (*p* < 0.001 for both comparisons).

**Figure 6:**
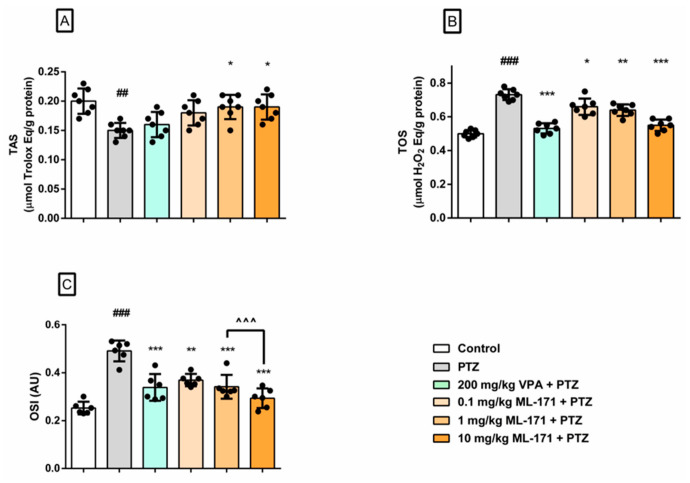
Effects of ML-171 on hippocampal oxidative stress parameters in PTZ-kindled rats. (**A**) Total antioxidant status (TAS), (**B**) total oxidant status (TOS), and (**C**) oxidative stress index (OSI) levels in hippocampal tissue. Data are presented as mean ± SD, and individual points represent separate animals. Statistical analysis was performed using one-way ANOVA followed by Tukey’s multiple comparisons test. ## *p* < 0.01 and ### *p* < 0.001 vs. the control group; * *p* < 0.05, ** *p* < 0.01, and *** *p* < 0.001 vs. the PTZ group. ^^^ *p* < 0.001 for the comparison between the 1 mg/kg ML-171 + PTZ and 10 mg/kg ML-171 + PTZ groups. PTZ, pentylenetetrazole; VPA, valproic acid; TAS, total antioxidant status; TOS, total oxidant status; OSI, oxidative stress index.

ML-171 treatment also modulated hippocampal oxidative stress parameters in a dose-related manner. TAS was not significantly increased by 0.1 mg/kg ML-171 (*p* > 0.05), whereas significant increases were observed at 1 and 10 mg/kg compared with the PTZ group (*p* < 0.05 for both comparisons). ML-171 significantly reduced TOS at 0.1 mg/kg (*p* < 0.05), 1 mg/kg (*p* < 0.01), and 10 mg/kg (*p* < 0.001) compared with PTZ alone. For OSI, significant reductions were observed at 0.1 mg/kg (*p* < 0.01), 1 mg/kg (*p* < 0.001), and 10 mg/kg (*p* < 0.001) compared with PTZ alone. In addition, OSI was significantly lower in the 10 mg/kg ML-171 group than in the 1 mg/kg ML-171 group (*p* < 0.001). Overall, these findings indicate that ML-171 attenuated PTZ-induced hippocampal oxidative imbalance, with a more consistent effect on oxidant load than on antioxidant capacity (Figure 6A–C).

#### 2.2.3. ML-171 Partially Attenuated PTZ-Induced Hippocampal Apoptosis-Related Signaling

**Figure 7:**
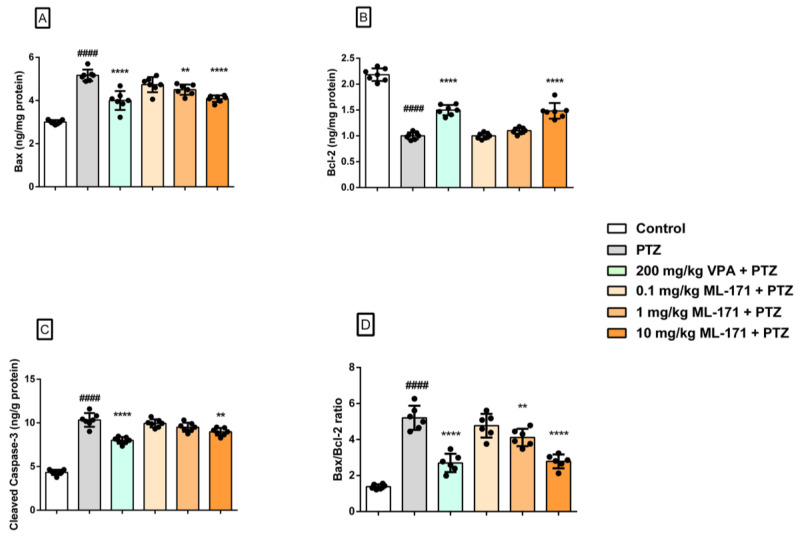
Effects of ML-171 on hippocampal apoptotic markers in PTZ-kindled rats. (**A**) Bax, (**B**) Bcl-2, (**C**) cleaved caspase-3, and (**D**) Bax/Bcl-2 ratio levels in hippocampal tissue. Data are presented as mean ± SD, and individual points represent separate animals. #### *p* < 0.0001 vs. the control group; ** *p* < 0.01, and **** *p* < 0.0001 vs. the PTZ group. PTZ, pentylenetetrazole; VPA, valproic acid; Bax, Bcl-2-associated X protein; Bcl-2, B-cell lymphoma 2.

PTZ administration induced a marked pro-apoptotic shift in hippocampal tissue, as indicated by significantly increased Bax (*p* < 0.0001), cleaved caspase-3 (*p* < 0.0001), and Bax/Bcl-2 ratio (*p* < 0.0001), together with decreased Bcl-2 levels (*p* < 0.0001) compared with the control group. Valproic acid significantly reversed all four PTZ-induced alterations (*p* < 0.0001 for each comparison vs. PTZ). The 0.1 mg/kg ML-171 dose did not significantly alter any of the evaluated apoptotic markers compared with PTZ alone (*p* > 0.05 for all comparisons). In contrast, 1 mg/kg ML-171 significantly reduced Bax levels and the Bax/Bcl-2 ratio (*p* < 0.01 for both comparisons), but did not significantly alter Bcl-2 or cleaved caspase-3 levels (*p* > 0.05). Treatment with 10 mg/kg ML-171 significantly reduced Bax (*p* < 0.0001), cleaved caspase-3 (*p* < 0.01), and the Bax/Bcl-2 ratio (*p* < 0.0001) and increased Bcl-2 levels (*p* < 0.0001) compared with the PTZ group. Overall, these findings suggest that ML-171 partially attenuated PTZ-induced hippocampal apoptosis-related signaling, with the most consistent biochemical effect observed at 10 mg/kg (Figure 7A–D).

#### 2.2.4. ML-171 Showed Endpoint- and Dose-Dependent Effects on PTZ-Induced Hippocampal Histopathological Injury

**Figure 8:**
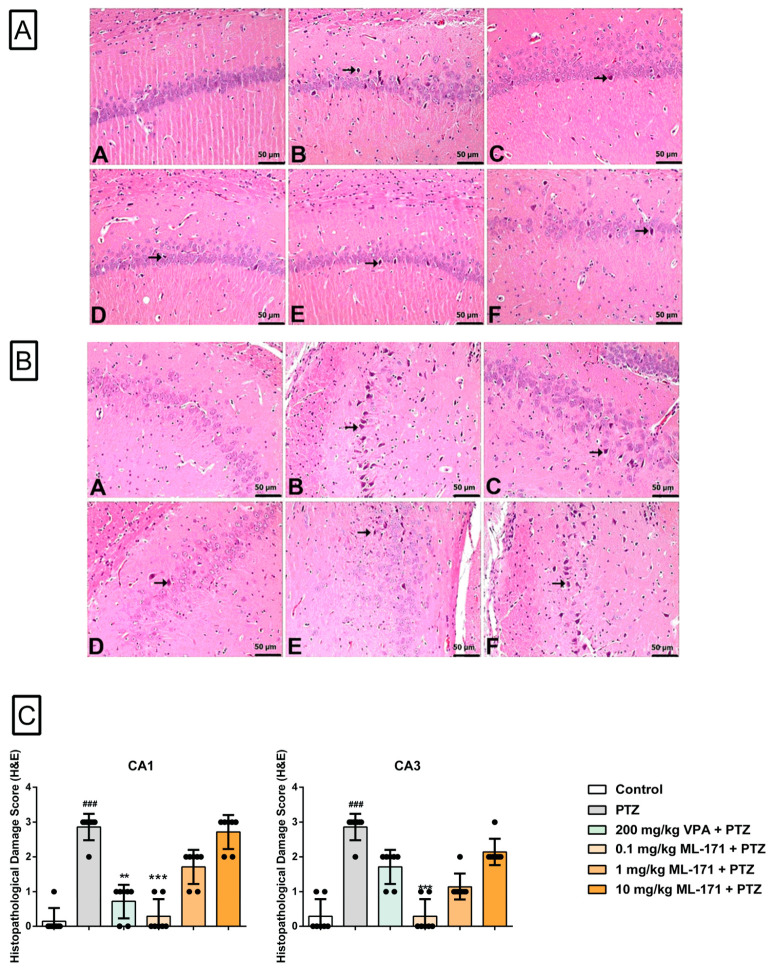
Histopathological changes in the CA1 and CA3 regions of the hippocampus in PTZ-kindled rats. (**A**,**B**) Representative hematoxylin and eosin staining in the CA1 and CA3 regions, respectively. In both panels: (A), control; (B), PTZ; (C), 200 mg/kg VPA + PTZ; (D), 0.1 mg/kg ML-171 + PTZ; (E), 1 mg/kg ML-171 + PTZ; and (F), 10 mg/kg ML-171 + PTZ. (**C**) Semiquantitative histopathological injury scores in the CA1 and CA3 regions. Data are presented as median [IQR], with individual points representing separate animals. Ordinal semiquantitative scores were analyzed using the Kruskal–Wallis test followed by Dunn’s multiple-comparisons test. Scale bar = 50 µm. Black arrows indicate pyknotic and degenerative neurons. ### *p* < 0.001 vs. the control group; ** *p* < 0.01 and *** *p* < 0.001 vs. the PTZ group. PTZ, pentylenetetrazole; VPA, valproic acid; CA1, cornu ammonis 1; CA3, cornu ammonis 3; IQR, interquartile range.

Histopathological evaluation showed that PTZ administration induced marked neuronal injury in both the CA1 and CA3 regions of the hippocampus compared with the control group. In the CA1 region, PTZ significantly increased histopathological injury scores compared with the control group (*p* < 0.001), whereas VPA (*p* < 0.01) and 0.1 mg/kg ML-171 (*p* < 0.001) significantly reduced these scores compared with the PTZ group. The reductions observed with 1 and 10 mg/kg ML-171 did not reach statistical significance (*p* > 0.05). In the CA3 region, PTZ significantly increased histopathological injury scores compared with the control group (*p* < 0.001). Treatment with 0.1 mg/kg ML-171 significantly reduced the injury score compared with the PTZ group (*p* < 0.001), whereas the reductions observed with VPA, 1 mg/kg ML-171, and 10 mg/kg ML-171 did not reach statistical significance (*p* > 0.05). Overall, these findings indicate that ML-171 attenuated PTZ-induced hippocampal histopathological injury mainly at the lowest tested dose, whereas higher doses did not produce parallel morphological protection despite biochemical improvements (Figure 8A–C).

#### 2.2.5. ML-171 Differentially Modulated PTZ-Induced NOX-1 Immunoreactivity in the Hippocampal CA1 and CA3 Regions

**Figure 9:**
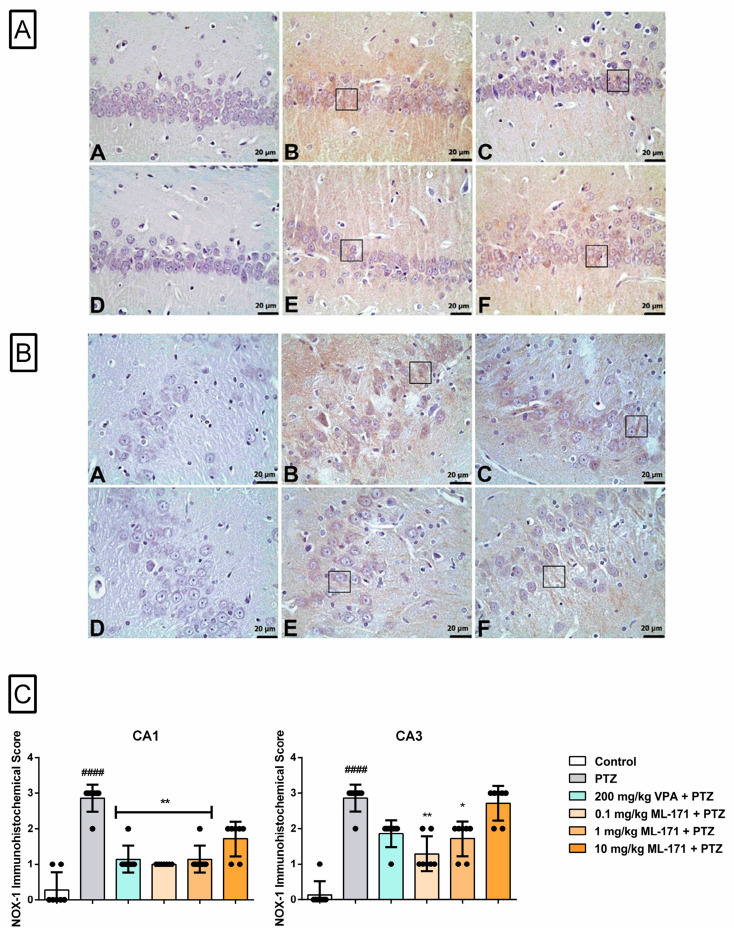
Effects of ML-171 on NOX-1 immunoreactivity in the CA1 and CA3 regions of the hippocampus in PTZ-kindled rats. (**A**,**B**) Representative NOX-1 immunohistochemical staining in the CA1 and CA3 regions, respectively. In both panels: (A), control; (B), PTZ; (C), 200 mg/kg VPA + PTZ; (D), 0.1 mg/kg ML-171 + PTZ; (E), 1 mg/kg ML-171 + PTZ; and (F), 10 mg/kg ML-171 + PTZ. Boxed areas indicate representative NOX-1-immunopositive regions. Scale bar = 20 µm. (**C**) Semiquantitative NOX-1 immunohistochemical scores in the CA1 and CA3 regions. Data are presented as median [IQR], with individual points representing separate animals. Ordinal semiquantitative scores were analyzed using the Kruskal–Wallis test followed by Dunn’s multiple-comparisons test. In the CA1 panel, the horizontal bracket indicates that the VPA, 0.1 mg/kg ML-171, and 1 mg/kg ML-171 groups each differed from the PTZ group (** *p* < 0.01). #### *p* < 0.0001 vs. the control group; * *p* < 0.05 and ** *p* < 0.01 vs. the PTZ group. PTZ, pentylenetetrazole; VPA, valproic acid; NOX-1, NADPH oxidase 1; CA1, cornu ammonis 1; CA3, cornu ammonis 3; IQR, interquartile range.

Immunohistochemical analysis revealed that PTZ administration markedly increased NOX-1 immunoreactivity in both the CA1 and CA3 regions of the hippocampus compared with the control group (*p* < 0.0001 for both regions). In the CA1 region, VPA, 0.1 mg/kg ML-171, and 1 mg/kg ML-171 significantly reduced NOX-1 immunoreactivity compared with PTZ alone (*p* < 0.01 for each comparison), whereas 10 mg/kg ML-171 did not produce a significant reduction (*p* > 0.05). In the CA3 region, 0.1 mg/kg ML-171 (*p* < 0.01) and 1 mg/kg ML-171 (*p* < 0.05) significantly reduced NOX-1 immunoreactivity compared with PTZ alone, whereas VPA and 10 mg/kg ML-171 did not show statistically significant reductions (*p* > 0.05). Overall, these findings indicate that ML-171 differentially modulated PTZ-induced hippocampal NOX-1 immunoreactivity in a region- and dose-dependent manner. Importantly, NOX-1 immunoreactivity should be interpreted as a regional protein-staining readout rather than as a direct measure of NOX-1 enzymatic activity (Figure 9A–C).

## 3. Discussion

The present study identifies a biologically relevant dissociation between seizure suppression and seizure-associated neuronal injury. ML-171 attenuated PTZ-induced oxidative and apoptotic injury in SH-SY5Y neuronal-like cells and in hippocampal tissue, but these injury-related effects were not accompanied by robust improvement in behavioral or electrophysiological seizure expression in PTZ-kindled rats. This pattern should not be interpreted solely as a negative antiseizure finding. Rather, it suggests that NOX-1-associated redox signaling may contribute more prominently to secondary neuronal injury triggered by seizure activity than to the acute network mechanisms responsible for seizure generation itself. Therefore, the present data support the interpretation that ML-171 acts primarily as an injury-modifying intervention in this model, rather than as a conventional antiseizure agent comparable to valproic acid.

PTZ successfully induced the expected pathological phenotype in both experimental arms of the study. In SH-SY5Y cells, PTZ markedly reduced cell viability, increased oxidant burden, and shifted apoptosis-related markers toward a pro-apoptotic profile. In the in vivo model, repeated PTZ administration produced severe seizure activity, epileptiform spike discharges, hippocampal redox imbalance, apoptotic signaling, and histopathological injury in the CA1 and CA3 regions. These findings are consistent with the established ability of PTZ to impair GABAergic inhibition, increase neuronal excitability, and activate oxidative and apoptotic cascades in experimental seizure models [6,26]. Therefore, the effects of ML-171 were evaluated within biologically active PTZ-induced injury models, allowing the distinction between effects on seizure activity and effects on seizure-associated cellular injury.

In SH-SY5Y cells, ML-171 showed a protective but non-linear concentration-response profile. Low-to-intermediate concentrations attenuated PTZ-induced loss of cell viability, whereas the highest concentration tested failed to confer significant protection. Moreover, ML-171 alone significantly reduced cell viability only at 160 µM, whereas 80–10 µM did not significantly affect basal viability. These findings indicate that ML-171 does not produce a simple monotonic dose-dependent protective effect in this model. Instead, its beneficial activity appears to occur within a restricted pharmacological window. The selection of 40 µM for subsequent mechanistic experiments was therefore justified, as this concentration combined clear protection against PTZ-induced cytotoxicity with an absence of measurable toxicity under basal conditions.

The loss of protection at 160 µM should not be regarded as invalidating the protective effect of ML-171. Rather, it points to an important pharmacological limitation. NOX-derived reactive oxygen species are not exclusively pathological; they also participate in physiological redox signaling, adaptive cellular responses, and intracellular communication [27,28]. Excessive suppression of redox-generating pathways, or concentration-dependent off-target effects, may therefore compromise cellular homeostasis rather than restore it [29,30]. This point is particularly relevant for small-molecule NOX inhibitors, whose biological effects may vary according to concentration, cell type, redox state, and experimental readout [15,31,32]. Thus, the in vitro viability data support the existence of a concentration-dependent therapeutic window for ML-171, not uniform protection across increasing exposure levels.

The oxidative stress findings further clarify the likely nature of this protection. PTZ significantly decreased TAS and markedly increased TOS and OSI in SH-SY5Y cells. ML-171 did not significantly restore TAS compared with PTZ alone, but it substantially reduced TOS and OSI. A similar pattern emerged in hippocampal tissue, where the effect of ML-171 was more consistent on oxidant burden than on antioxidant capacity. This distinction is mechanistically meaningful. If ML-171 primarily acted by globally strengthening antioxidant defenses, a more prominent restoration of TAS would be expected. Instead, the dominant effect was reduction of oxidant load. Given that NOX enzymes are dedicated ROS-generating systems, this pattern is compatible with attenuation of oxidant production rather than direct enhancement of antioxidant reserve [33,34]. Nevertheless, TAS, TOS, and OSI are global redox indices and cannot identify the precise enzymatic or subcellular source of ROS [35]. Therefore, these findings are compatible with, but do not directly prove, NOX-1-associated suppression of ROS generation.

The apoptosis-related data are consistent with the redox findings but should not be interpreted as establishing a direct causal sequence between reduced oxidant burden and suppression of apoptotic signaling. PTZ increased Bax and cleaved caspase-3 levels, decreased Bcl-2 levels, and elevated the Bax/Bcl-2 ratio in SH-SY5Y cells, indicating a shift toward a pro-apoptotic profile [36,37,38]. ML-171 pretreatment was associated with attenuation of these apoptosis-related alterations, including lower Bax, cleaved caspase-3, and Bax/Bcl-2 ratio values and higher Bcl-2 levels compared with PTZ-treated cells. Flow cytometry further supported this pattern, as PTZ reduced the viable-cell population and increased early and late apoptotic fractions, whereas ML-171 pretreatment was associated with preservation of the viable-cell population and reduction of apoptotic fractions. Importantly, the dead-cell fraction was not significantly modified by ML-171. Taken together, the XTT, TAS/TOS/OSI, ELISA, and Annexin V-based flow cytometry findings support an association between reduced oxidant burden and attenuated apoptosis-related injury in PTZ-exposed SH-SY5Y neuronal-like cells; however, they do not prove that oxidative stress reduction directly caused the observed changes in apoptotic signaling.

Although these findings support an association between reduced oxidant burden and attenuation of apoptosis-related signaling, the molecular link between NOX-1-associated redox modulation and cell-fate regulation is likely to involve mechanisms beyond the classical Bax/Bcl-2/caspase-3 axis evaluated here. Apoptotic and injury-related responses can also be shaped upstream by host regulatory factors, stress-responsive RNA processing, and cell-state remodeling. In an *M. bovis*-infected epithelial model, Wang et al. showed that the RNA-binding protein RBMX2 promoted apoptosis by reducing APAF-1 intron retention, illustrating how alternative RNA processing can regulate apoptotic susceptibility [39]. In a subsequent study, RBMX2 was also linked to infection-associated epithelial–mesenchymal transition and lung cancer progression, providing a broader example of how persistent cellular stress and host–cell reprogramming may contribute to pathological tissue remodeling [40]. Although these mechanisms were identified in infection-related epithelial systems and cannot be directly extrapolated to PTZ-exposed neuronal cells, they emphasize that Bax, Bcl-2, cleaved caspase-3, and Annexin V represent downstream readouts within a broader regulatory network. Therefore, the present findings should be interpreted as evidence of attenuated apoptosis-related injury rather than as a complete mechanistic explanation of redox-regulated cell death.

The in vivo seizure data provide the key contrast underlying this interpretation. Valproic acid, as expected, reduced seizure severity, prolonged first myoclonic jerk latency, and decreased epileptiform spike number. In contrast, ML-171 did not significantly improve modified Racine score, first myoclonic jerk latency, spike number, or GTCS duration at the tested doses. Although 10 mg/kg ML-171 tended to prolong GTCS onset latency, this effect did not remain significant after correction for multiple comparisons. These findings indicate that ML-171 does not behave as a robust antiseizure agent under the present experimental conditions. Importantly, however, this limited effect on seizure expression occurred despite improvement in several oxidative, apoptotic, and histological injury-related endpoints. Thus, the limited effect on seizure expression should be interpreted alongside the attenuation of oxidative, apoptotic, and selected histological injury-related endpoints, rather than as evidence of direct antiseizure activity.

This dissociation is biologically plausible because acute seizure expression and seizure-associated neuronal injury are regulated by overlapping but non-identical mechanisms. Behavioral and electrophysiological seizure activity is largely determined by network excitability, inhibitory-excitatory balance, ion channel function, and circuit synchronization. In contrast, seizure-associated tissue injury involves secondary downstream processes such as oxidant production, mitochondrial stress, lipid and protein oxidation, inflammatory amplification, and apoptosis-related signaling [41]. Within this framework, NOX-1-associated redox signaling may be more closely linked to the injury cascade activated by recurrent seizures than to the immediate initiation of seizure discharges. Suppression of cellular stress responses may therefore attenuate pathological progression, neuronal remodeling, and cumulative tissue damage even when the primary proconvulsant stimulus and seizure phenotype are not fully suppressed. This interpretation is consistent with the present finding that ML-171 improved injury-related endpoints without producing VPA-like antiseizure efficacy.

The hippocampal biochemical findings support this injury-modifying interpretation. PTZ decreased TAS and increased TOS and OSI, confirming a marked redox imbalance after kindling and final PTZ challenge. ML-171 reduced TOS at all tested doses and decreased OSI, with the most pronounced OSI reduction observed at 10 mg/kg. TAS improvement was more modest and reached significance only at selected doses. This pattern parallels the cellular findings and reinforces the view that ML-171 preferentially reduces oxidant burden rather than uniformly restoring antioxidant capacity. In parallel, PTZ increased Bax, cleaved caspase-3, and the Bax/Bcl-2 ratio while decreasing Bcl-2 in hippocampal tissue [42,43,44]. ML-171 partially reversed these changes in a dose- and endpoint-dependent manner. The 0.1 mg/kg dose did not significantly improve the evaluated apoptotic markers, 1 mg/kg reduced Bax and the Bax/Bcl-2 ratio, and 10 mg/kg produced the most consistent anti-apoptotic profile. These findings indicate that ML-171 can attenuate hippocampal apoptosis-related signaling even when overt seizure parameters are not robustly improved.

A major complexity of the study is that biochemical, histopathological, and NOX-1 immunohistochemical outcomes did not follow a uniform dose–response pattern. In hippocampal biochemical assays, 10 mg/kg ML-171 produced the most consistent improvement in oxidative and apoptotic markers. In contrast, histopathological protection was most evident at 0.1 mg/kg, whereas 1 and 10 mg/kg did not significantly reduce CA1 or CA3 injury scores compared with PTZ. This discrepancy is important and indicates that the biological effects of ML-171 should be interpreted in an endpoint-specific rather than uniformly dose-dependent manner. Biochemical assays and histopathological scoring reflect different biological levels. TAS, TOS, OSI, Bax, Bcl-2, and cleaved caspase-3 are molecular readouts that may respond relatively rapidly to changes in redox and apoptotic signaling. Histopathological scoring, by contrast, captures the morphological consequence of injury at the tissue level and is influenced by regional vulnerability, neuronal subtype, sampling plane, fixation quality, timing of tissue collection, and the sensitivity of semiquantitative scoring [45,46,47].

Accordingly, improvement in soluble oxidative or apoptotic markers at a single postictal time point does not necessarily imply proportional improvement in visible tissue morphology. Repeated PTZ exposure may establish structural alterations that are less readily reversible than acute biochemical changes. Conversely, the prominent histological benefit observed at 0.1 mg/kg may indicate that lower-dose ML-171 more effectively preserved regional hippocampal morphology, even though its global biochemical effects were less pronounced. One possible explanation is that different ML-171 exposures may differentially affect pathological and physiological redox processes, although this remains speculative because tissue exposure and NOX activity were not directly measured [27,30]. At higher doses, broader pharmacological effects may reduce soluble oxidative/apoptotic markers without producing equivalent structural preservation. Thus, the in vivo response to ML-171 should be regarded as endpoint-dependent rather than uniformly dose-dependent.

The NOX-1 immunohistochemistry findings further support this interpretation. PTZ increased NOX-1 immunoreactivity in both CA1 and CA3 regions, supporting an association between PTZ-induced hippocampal injury and increased regional NOX-1 staining. ML-171 reduced NOX-1 immunoreactivity mainly at low-to-intermediate doses. In CA1, valproic acid, 0.1 mg/kg ML-171, and 1 mg/kg ML-171 significantly reduced NOX-1 immunoreactivity, whereas 10 mg/kg did not. In CA3, 0.1 and 1 mg/kg ML-171 reduced NOX-1 immunoreactivity, whereas valproic acid and 10 mg/kg did not reach statistical significance. Notably, the doses that most clearly reduced NOX-1 immunoreactivity were also more closely aligned with the histopathological benefit, while 10 mg/kg improved several biochemical parameters without parallel improvement in NOX-1 immunoreactivity or histological scores.

This pattern argues against a simplistic model in which increasing ML-171 dose progressively suppresses NOX-1 expression, reduces oxidative stress, prevents apoptosis, and preserves tissue morphology. Immunohistochemistry measures regional protein immunoreactivity and staining intensity; it does not measure enzymatic activity [47]. Changes in enzymatic ROS generation or putative target engagement may occur without a proportional change in immunohistochemical staining, while reduced immunoreactivity does not quantify the degree of enzyme inhibition. Therefore, the antioxidant and anti-apoptotic effects observed at 10 mg/kg cannot be attributed solely to reduced NOX-1 expression or directly proven NOX-1 inhibition. They may reflect NOX-1-associated redox modulation, modulation of other ROS-generating systems, compensatory changes among NOX isoforms, altered mitochondrial ROS production, or secondary effects on apoptosis-related signaling [34,35,48]. For this reason, the most defensible conclusion is that ML-171 differentially modulated PTZ-induced NOX-1 immunoreactivity and attenuated seizure-associated oxidative/apoptotic injury in a region- and endpoint-dependent manner.

The dose–response pattern observed in the in vivo experiments was not strictly monotonic and should therefore be interpreted as endpoint-dependent rather than as a simple linear pharmacodynamic response. Biochemical markers of oxidative stress and apoptosis tended to show more consistent improvement at higher ML-171 doses, particularly at 10 mg/kg, whereas histopathological protection was more evident at the lowest dose tested. This discrepancy may reflect several non-mutually exclusive mechanisms. First, biochemical measurements were performed in whole hippocampal tissue homogenates and may capture global redox and apoptotic changes, whereas histopathological and immunohistochemical scores reflect regional and cellular-level alterations within CA1 and CA3. Second, hippocampal subregions may differ in their vulnerability to PTZ-induced injury, NOX isoform contribution, compensatory antioxidant responses, and sensitivity to ML-171. Third, excessive or sustained suppression of redox signaling at higher doses may not necessarily translate into better morphological preservation, because ROS also participate in adaptive cellular signaling. Finally, NOX-1 immunoreactivity reflects regional protein staining rather than enzymatic activity and may therefore not parallel biochemical oxidative stress indices in a direct one-to-one manner. Thus, the present findings support a non-linear, region-dependent, and endpoint-specific pharmacological profile of ML-171 in PTZ-kindled rats.

The pharmacological properties of ML-171 should also be considered when interpreting these non-linear findings. ML-171 is a small-molecule NOX-1 inhibitor, but the biological behavior of NOX inhibitors is influenced by concentration, solubility, formulation, tissue exposure, and potential off-target actions [15,32]. Because ML-171 concentrations in plasma or brain tissue were not measured, the relationship between administered dose, target exposure, putative NOX-1 engagement, and biological response cannot be directly established in the present study. In the present study, ML-171 required DMSO-based preparation and dilution in corn oil for in vivo administration, and matched vehicle conditions were used to reduce vehicle-related confounding. Nevertheless, brain exposure, metabolic stability, tissue distribution, and systemic tolerability were not directly assessed. The concentration-dependent cellular injury observed in vitro, together with the divergence between high-dose biochemical protection and limited histological/IHC benefit in vivo, suggests that ML-171 has a restricted pharmacological window. Therefore, the present data should not be interpreted as evidence that higher ML-171 exposure necessarily produces greater attenuation of seizure-associated injury. Rather, they indicate that ML-171 can reduce selected biochemical markers of injury, while its structural and regional effects may depend on dose, tissue exposure, and the biological endpoint examined.

The biological relevance of this dissociation may extend beyond the acute postictal period. Recurrent seizure activity can activate stress-responsive pathways that influence not only immediate oxidative and apoptotic injury, but also longer-term cellular phenotypes, synaptic remodeling, glial responses, and progressive hippocampal vulnerability. Within this context, attenuation of oxidant burden and apoptosis-related signaling may be meaningful even in the absence of robust acute seizure suppression, because secondary injury cascades may contribute to tissue remodeling and disease progression after repeated seizures. However, the present study was not designed to evaluate these long-term consequences. Spontaneous seizure recurrence, chronic neuronal remodeling, gliosis, cognitive outcomes, and delayed histopathological progression were not assessed. Therefore, future longitudinal studies are required to determine whether NOX-1-associated redox modulation can influence the longer-term structural and functional outcomes of recurrent seizure activity.

The comparison with valproic acid further clarifies the pharmacological and translational positioning of ML-171. Valproic acid improved behavioral and electrophysiological seizure parameters and also ameliorated oxidative and apoptotic alterations, consistent with its established antiseizure efficacy and its reported effects on oxidative stress, mitochondrial function, and apoptosis-related pathways in experimental neuronal injury models [25,42,49]. ML-171, in contrast, did not match valproic acid in suppressing seizure expression, but it improved several biochemical and selected histological indices of hippocampal injury. Therefore, the present data do not support ML-171 as an alternative or substitute for established antiseizure therapy. A more appropriate translational interpretation is that pharmacological modulation of NOX-1-associated redox pathways may represent an adjunctive injury-modifying strategy aimed at limiting seizure-associated oxidative and apoptotic damage while conventional antiseizure medications control neuronal hyperexcitability and seizure expression [50,51]. Such an approach may be particularly relevant in conditions characterized by recurrent seizures, cumulative oxidative stress, and progressive hippocampal injury; however, this remains a preclinical interpretation that requires validation using chronic treatment protocols, long-term functional and histological outcomes, pharmacokinetic assessment, and direct measurements of NOX activity.

Several limitations should be acknowledged. First, VPA and ML-171 were administered acutely 60 min before the final PTZ challenge after completion of the kindling acquisition period and were not administered during the development of kindling. Therefore, the present design primarily evaluates acute modulation of seizure expression and seizure-associated neuronal injury in fully kindled animals. It does not allow assessment of whether NOX-1-targeted modulation influences epileptogenesis or the acquisition and progression of kindling. Accordingly, the present findings should not be directly extrapolated to chronic antiseizure efficacy or disease-modifying effects. Second, NOX-1 enzymatic activity was not directly measured. Semiquantitative NOX-1 immunohistochemistry provides a regional protein-staining readout that reflects relative immunoreactivity and tissue distribution, rather than catalytic activity or pharmacological target engagement. Therefore, reductions in NOX-1 staining cannot be interpreted as direct evidence of reduced NOX-1 enzymatic activity. Accordingly, the present study cannot distinguish whether the observed effects were due to direct NOX-1 inhibition, altered NOX-1 expression, modulation of other NOX isoforms, changes in mitochondrial ROS generation, or downstream effects on apoptosis-related signaling. The observed reductions in TOS, OSI, and apoptosis-related markers are consistent with NOX-1-associated redox modulation, but they do not establish that these effects resulted exclusively from direct inhibition of NOX-1 activity in hippocampal tissue. Third, other NOX isoforms, particularly NOX-2 and NOX-4, were not assessed. This is relevant because seizure-induced oxidative stress may involve multiple NOX isoforms with distinct cellular sources, temporal profiles, and functions [52,53]. Fourth, TAS, TOS, and OSI are global redox indicators and do not identify specific ROS species, subcellular ROS sources, or oxidative damage products [34,54]. Fifth, ML-171 pharmacokinetics, brain penetration, metabolic stability, and systemic toxicity were not evaluated; therefore, the optimal therapeutic exposure range cannot be defined from the present data. Sixth, SH-SY5Y cells are useful for modeling neuronal-like oxidative and apoptotic injury; however, unless differentiated, they do not fully reproduce the phenotype of mature neurons or the complexity of hippocampal neuronal networks [36,55]. Seventh, only adult male rats were used; therefore, possible sex-dependent differences in seizure susceptibility, NOX-related redox signaling, and ML-171 responsiveness could not be addressed [52,56]. Eighth, biochemical, histopathological, and immunohistochemical assessments were performed at a single postictal time point. Because oxidative stress, apoptotic signaling, NOX-1 immunoreactivity, and tissue morphology may follow different temporal trajectories after repeated PTZ exposure, time-course studies are needed to determine whether the divergence between biochemical and histological outcomes persists or changes over time [57,58]. In addition, the relatively small final group size should be considered when interpreting negative behavioral and electrophysiological findings, particularly for endpoints with high inter-animal variability. Finally, histopathological and immunohistochemical scores are semiquantitative ordinal measures and should not be interpreted as continuous molecular readouts [46].

Despite these limitations, our study has several strengths. It integrates cellular and animal models, combines behavioral and electrophysiological seizure assessment with biochemical, flow cytometric, histopathological, and immunohistochemical endpoints, and includes valproic acid as a pharmacological reference. This multi-level design allowed us to distinguish between effects on seizure expression and effects on seizure-associated secondary neuronal injury. The resulting pattern is internally coherent: ML-171 showed limited antiseizure activity but attenuated oxidative and apoptotic injury, particularly at the biochemical level. The lack of a uniform dose–response across all endpoints does not weaken the study if interpreted appropriately; rather, it highlights the biological complexity of NOX-1–associated redox modulation in epilepsy-related neuronal injury.

## 4. Materials and Methods

### 4.1. Study Design and Reporting

This study combined in vitro experiments in SH-SY5Y neuronal-like cells with an in vivo PTZ-kindling rat model to evaluate whether ML-171 modulates PTZ-associated oxidative and apoptosis-related injury and whether these effects are accompanied by changes in behavioral and electrocorticographic seizure parameters. In vitro experiments were performed as independent biological experiments with technical replicates. In vivo outcomes included behavioral seizure scoring, ECoG analysis, hippocampal biochemical assays, histopathology, and NOX-1 immunohistochemistry. Where applicable, outcome assessment was performed using coded samples to reduce observer bias.

### 4.2. In Vitro Studies

#### 4.2.1. Cell Culture, Reagents, and Experimental Design

Human neuroblastoma SH-SY5Y cells (American Type Culture Collection [ATCC], Manassas, VA, USA; CRL-2266) were used as a neuronal-like cellular model for the in vitro experiments. Cells were maintained in DMEM/F-12 (1:1; Thermo Fisher Scientific, Altrincham, UK) supplemented with 10% fetal bovine serum, 1% L-glutamine, and 1% penicillin-streptomycin (10,000 U/mL), all obtained from Sigma-Aldrich Co. (St. Louis, MO, USA) at 37 °C in a humidified incubator with 5% CO_2_. Cells were passaged at approximately 80% confluence using phosphate-buffered saline and trypsin-EDTA (Sigma-Aldrich Co., St. Louis, MO, USA). Cells were not differentiated before treatment.

Pentylenetetrazole (PTZ; Sigma-Aldrich Co., St. Louis, MO, USA), sodium valproate (VPA; Sigma-Aldrich Co., St. Louis, MO, USA), and a pharmacological NOX-1 inhibitor ML-171 (MedChemExpress, Monmouth Junction, NJ, USA; Cat. No. HY-12805) were used as experimental agents. PTZ and VPA were freshly prepared in culture medium before each experiment. ML-171 was initially dissolved in dimethyl sulfoxide (DMSO) to prepare stock solutions and subsequently diluted in culture medium to the desired final concentrations. The final DMSO concentration in all ML-171-containing wells was kept below 0.1% (*v*/*v*), and the same final DMSO concentration was applied to the corresponding control and PTZ-only wells to maintain matched vehicle conditions.

To evaluate the potential cytoprotective effect of ML-171 against PTZ-induced neuronal injury, SH-SY5Y cells were divided into six groups: control, PTZ, ML-171, ML-171 + PTZ, VPA, and VPA + PTZ. The concentration ranges of ML-171 and VPA were determined based on previously published in vitro studies, relevant literature data, and preliminary experiments conducted in our laboratory. Accordingly, ML-171 was tested at 10, 20, 40, 80 µM and 160 µM, whereas VPA was tested at 0.5, 1, and 2 mM. Cells in the PTZ group were exposed to 30 mM PTZ for 24 h. Cells in the ML-171-only and VPA-only groups were treated with ML-171 or VPA, respectively, for 24 h. In the PTZ + ML-171 and PTZ + VPA groups, cells were pretreated with ML-171 or VPA for 3 h, respectively, and then exposed to 30 mM PTZ for 24 h without removal of the pretreatment medium. Thus, ML-171 or VPA remained present throughout the subsequent PTZ exposure period. After incubation, the medium was removed, and the wells were washed before performing the XTT assay [25,59,60].

Based on the XTT viability screening, 40 µM ML-171 was selected for subsequent in vitro biochemical and flow cytometric experiments. This concentration was chosen because it provided robust protection against PTZ-induced cytotoxicity while showing no detectable reduction in basal cell viability when administered alone. Although 80 µM ML-171 also attenuated PTZ-induced cytotoxicity, 40 µM was preferred as a more conservative concentration with a clearer tolerability margin for subsequent experiments.

#### 4.2.2. XTT Cell Viability Assay

Cell viability was assessed using an XTT assay kit (Biological Industries, Kibbutz Beit-Haemek, Israel). SH-SY5Y cells were seeded into 96-well plates at a density of approximately 1 × 10^4^ cells/well and allowed to attach for 24 h. Following treatment, 50 µL XTT solution was added to each well containing phenol red-free medium, and the plates were incubated for 4 h at 37 °C. Absorbance was measured at 450 nm using a microplate reader (Thermo Fisher Scientific, Altrincham, UK). The XTT assay was performed in three independent biological experiments, each including three technical replicates per group. Technical replicates were averaged within each independent experiment, and the independent experiment mean was used as the statistical unit. Cell viability was calculated as follows: cell viability (%) = (OD of treated cells/OD of control cells) × 100.

#### 4.2.3. Measurement of Total Antioxidant Status (TAS) and Total Oxidant Status (TOS)

For TAS and TOS analyses, SH-SY5Y cells were seeded into 6-well plates at a density of approximately 5 × 10^5^ cells/well. Following treatment, cells were harvested, washed with PBS, adjusted to approximately 1 × 10^6^ cells/mL, and subjected to three repeated freeze–thaw cycles to disrupt cellular membranes. After centrifugation at 4000 rpm for 10 min at 4 °C, the supernatants were collected for biochemical analyses. TAS and TOS levels were measured using commercial colorimetric kits (Rel Assay Diagnostics, Gaziantep, Türkiye). TAS values were expressed as mmol Trolox equivalent/mg protein, whereas TOS values were expressed as µmol H_2_O_2_ equivalent/mg protein [61,62]. Total protein concentrations were determined using a Bradford protein assay kit (Merck Millipore, Darmstadt, Germany). The oxidative stress index (OSI) was calculated from protein-normalized TOS and TAS values after unit harmonization. For this calculation, TAS values were converted from mmol Trolox equivalent/mg protein to µmol Trolox equivalent/mg protein, and OSI was calculated using the following formula: OSI = [(TOS, µmol H_2_O_2_ equivalent/mg protein) / (TAS, µmol Trolox equivalent/mg protein)] × 100.

#### 4.2.4. ELISA Analysis of Apoptosis-Related Proteins

Bax, Bcl-2, and cleaved caspase-3 levels were measured in SH-SY5Y cell lysates using commercial human ELISA kits according to the manufacturers’ instructions. The kits used were Bcl-2 (ELK Biotechnology, Wuhan, China; Cat. No. ELK10289), Bax (ELK Biotechnology, Wuhan, China; Cat. No. ELK1532), and cleaved caspase-3 (BT Laboratory, Shanghai, China; Cat. No. E4804Hu). Briefly, cells were collected after treatment, washed with PBS, and lysed by repeated freeze–thaw cycles. Absorbance values were recorded using a microplate reader, and concentrations were calculated from the corresponding standard curves. The Bax/Bcl-2 ratio was calculated for each sample using protein-normalized Bax and Bcl-2 concentrations. All ELISA data were normalized to total protein content.

#### 4.2.5. Total Protein Assay

Total protein concentrations were determined by the Bradford method for normalization of TAS, TOS, and ELISA findings. Briefly, bovine serum albumin standards were prepared by serial dilution, and 10 µL of standards or samples were loaded into 96-well plates in duplicate. After addition of 200 µL Bradford reagent, absorbance was measured at 595 nm, and protein concentrations were calculated using the standard curve [63].

#### 4.2.6. Flow Cytometric Analysis of Apoptosis

Apoptosis was evaluated using a Muse Annexin V & Dead Cell Assay Kit (Merck Millipore, Darmstadt, Germany; Cat. No. MCH100105) and a Muse Cell Analyzer (EMD Millipore Corporation, Billerica, MA, USA) [64]. SH-SY5Y cells were seeded into 6-well plates and treated with 40 µM ML-171 or 1 mM VPA for 3 h before exposure to 30 mM PTZ for 24 h. After treatment, both floating and adherent cells were collected, washed with PBS containing 1% fetal bovine serum, and stained according to the manufacturer’s protocol. Viable, early apoptotic, late apoptotic, and necrotic cell populations were quantified using the Muse Cell Analyzer.

### 4.3. In Vivo Studies

#### 4.3.1. Animals and Ethical Approval

Adult male Wistar Albino rats (4 months old, 230–250 g) were used in the in vivo experiments. The animals were obtained from the Experimental Animals Laboratory of Sivas Cumhuriyet University Faculty of Medicine and housed under standard laboratory conditions in a sound-insulated room maintained at 22 ± 1 °C and 55 ± 6% relative humidity, under a 12-h light/12-h dark cycle, with free access to standard chow and water. To minimize circadian variability, all experimental procedures were performed between 09:00 and 12:00 under standardized light and sound conditions. The experimental protocol was approved by the Local Ethics Committee for Animal Experiments of Sivas Cumhuriyet University (approval date: 5 January 2024; decision no: 13).

#### 4.3.2. Experimental Groups and PTZ-Kindling Protocol

The study was initially planned with 48 rats. During the PTZ-kindling period, four animals were lost because of severe seizures, one animal was excluded because it failed to reach the kindling criterion, and one animal was excluded because of technical failure during sample collection or recording; therefore, the final analyses were performed with 42 rats, with seven animals in each group. The animals were allocated into six groups: control, PTZ, VPA + PTZ, 0.1 mg/kg ML-171 + PTZ, 1 mg/kg ML-171 + PTZ, and 10 mg/kg ML-171 + PTZ.

To establish the experimental epilepsy model, all groups except the control group were subjected to a subacute PTZ-kindling protocol. PTZ (35 mg/kg; Sigma-Aldrich Co., St. Louis, MO, USA) was dissolved in physiological saline and administered intraperitoneally at a volume of 1 mL/kg, three times per week for 33 days, for a total of 15 injections. After each injection, the animals were placed individually in transparent Plexiglas cages (50 × 40 × 40 cm) and observed for 30 min. Seizure severity was scored using a modified Racine scale as follows: 0, no response; 1, facial and ear twitching; 2, myoclonic jerks and head nodding; 3, unilateral forelimb clonus; 4, bilateral forelimb clonus with rearing; and 5, generalized tonic–clonic seizures with loss of postural control. Rats that displayed stage 5 seizures on three separate occasions were considered fully kindled. This modified Racine scale was used throughout the kindling period and during the final PTZ challenge [65].

Sodium valproate (Sigma-Aldrich Co., St. Louis, MO, USA) was used as the positive control at a dose of 200 mg/kg (i.p.) and was freshly dissolved in saline before administration. ML-171 (MedChemExpress, Monmouth Junction, NJ, USA; Cat. No. HY-12805) was administered intraperitoneally at doses of 0.1, 1, and 10 mg/kg. ML-171 was first dissolved in DMSO and then diluted with corn oil immediately before injection to obtain the required final concentrations. The final DMSO content did not exceed 10%, and the injection volume did not exceed 1 mL/kg. VPA and ML-171 were administered 60 min before the final PTZ challenge and were not administered throughout the entire kindling acquisition period. To control for possible vehicle-related effects, rats in the control, PTZ, and VPA + PTZ groups also received the corresponding DMSO/corn oil vehicle as a sham pretreatment at the same time point and injection volume used in the ML-171 groups. On the final experimental day, PTZ was re-administered at 35 mg/kg (i.p.), and synchronized electrocorticographic (ECoG) and video recordings were obtained for 30 min. The use of 200 mg/kg sodium valproate as a positive control is consistent with prior PTZ-kindling rat studies [66].

#### 4.3.3. Stereotaxic Surgery and Electrode Implantation

All experimental animals, including kindled rats and non-kindled controls, underwent electrode implantation for electrophysiological recording. General anesthesia was induced with ketamine (90 mg/kg, i.p.) and xylazine (10 mg/kg, i.p.). Adequacy of anesthesia was verified by loss of the corneal and paw withdrawal reflexes. After shaving the cranial region and antiseptic preparation with povidone-iodine, the rats were fixed in a stereotaxic frame, and the skull was exposed through a midline scalp incision of approximately 3 cm.

Electrode coordinates were determined according to the rat brain atlas of Paxinos and Watson [67]. The positive electrode was placed 4 mm anterior to bregma and 3 mm right lateral to the midline; the negative electrode was placed 4 mm posterior to bregma and 3 mm right lateral to the midline; and the ground electrode was placed 4 mm posterior to bregma and 3 mm left lateral to the midline. Burr holes were drilled at the designated coordinates, stainless-steel screw electrodes were positioned so as to contact the cranial surface, and the leads were secured with dental acrylic. After surgery, the animals were allowed to recover for 1 week. Postoperative monitoring was performed daily during this period. To reduce the risk of postoperative infection, sultamicillin (50 mg/kg, i.p.) was administered twice daily for 3 days. The final PTZ challenge and synchronized ECoG/video recording were performed after completion of the 1-week recovery period.

#### 4.3.4. ECoG Recordings and Electrophysiological Analysis

ECoG signals were amplified using a BioAmp amplifier and digitally acquired with a PowerLab 4/SP data acquisition system (ADInstruments Pty Ltd., Bella Vista, NSW, Australia). The recordings were analyzed using LabChart v7.0.3 software (ADInstruments Pty Ltd., Bella Vista, NSW, Australia). Simultaneous video recording was performed throughout the 30-min acquisition period to enable synchronized behavioral and electrophysiological evaluation. To minimize low-frequency drift, high-frequency noise, and movement-related artifacts, ECoG recordings were digitally band-pass filtered between 1 and 34 Hz before analysis. Recording segments containing gross movement artifacts, signal saturation, electrode disconnection, or unstable baseline activity were excluded according to predefined visual criteria applied uniformly across all experimental groups.

For each animal, the basal ECoG activity threshold was determined individually. Epileptic spike activity was defined as rhythmic waveforms with an amplitude at least three times greater than baseline and showing progressive spike-frequency characteristics. The following parameters were analyzed: latency to the first myoclonic jerk (IMJ), epileptiform spike number per minute, calculated by dividing the total spike count during the 30-min recording period by 30, generalized tonic–clonic seizure (GTCS) onset latency, and GTCS duration. IMJ was determined by matching the first epileptic spike on ECoG with the first myoclonic movement observed in the synchronized video recording. GTCS onset was defined as the first concurrent appearance of tonic–clonic motor manifestations on video and spike-wave activity on ECoG. GTCS duration was calculated from seizure onset to the end of the tonic–clonic episode. Animals that did not develop GTCS during the 30-min recording period were assigned the maximum observation value of 1800 s for descriptive comparison; this limitation was considered when interpreting GTCS onset latency.

#### 4.3.5. Tissue Collection and Hippocampal Homogenization

Animals were sacrificed 24 h after the final PTZ challenge to capture early postictal NOX-1–related oxidative and apoptotic alterations together with acute hippocampal histopathological changes. Before tissue collection, cardiac perfusion with phosphate-buffered saline (PBS) was performed to remove blood from the cerebral circulation. The brains were rapidly removed on an ice-cold surface. For each animal, the right hippocampus was anatomically dissected and transferred into labeled sterile microtubes for biochemical analyses, whereas the contralateral hemisphere was fixed in 10% neutral buffered formalin for histopathological and immunohistochemical analyses.

Each hippocampal sample was weighed, and PBS (pH 7.4) was added at a 1:9 (*w*/*v*) ratio. The tissues were homogenized on ice using a manual blade homogenizer under cold conditions to minimize protein degradation. The homogenates were then centrifuged at 5000× *g* for 5 min at 4 °C, and the supernatants were carefully collected and stored at −80 °C until biochemical analyses.

#### 4.3.6. Determination of TAS and TOS in Hippocampal Tissue

Hippocampal total antioxidant status (TAS) and total oxidant status (TOS) were measured spectrophotometrically using commercial assay kits (Rel Assay Diagnostics, Gaziantep, Turkey) in accordance with the manufacturer’s instructions. TAS was determined based on the inhibition of ABTS radical formation by antioxidants present in the sample, and absorbance was read at 660 nm. TOS was determined by colorimetric measurement of oxidant-dependent ferrous ion oxidation, and absorbance was read at 530 nm. All measurements were performed in duplicate in 96-well microplates, and calibration and control procedures were carried out according to the manufacturer’s recommendations.

#### 4.3.7. ELISA Analysis of Hippocampal Apoptotic Markers

Hippocampal Bcl-2, Bax, and cleaved caspase-3 levels were measured in tissue supernatants using commercial rat ELISA kits (BT Laboratory, Shanghai, China): Bcl-2 (Cat. No. E0037Ra), Bax (Cat. No. E1869Ra), and cleaved caspase-3 (Cat. No. E1648Ra). Samples were assayed in duplicate in separate 96-well ELISA plates for each analyte, according to the manufacturers’ protocols. Concentrations were calculated from the corresponding standard curves and normalized to total protein content.

#### 4.3.8. Total Protein Assay

To normalize TAS, TOS, and ELISA results, total protein concentrations in hippocampal supernatants were determined by the Bradford method using a commercial protein assay kit (ABP Biosciences, Beltsville, MD, USA). Standard solutions were prepared using bovine serum albumin, and sample absorbance values were measured spectrophotometrically according to the manufacturer’s instructions.

#### 4.3.9. Histopathological and Immunohistochemical Analyses

For histopathological evaluation, brain tissues collected at necropsy were fixed in 10% neutral buffered formalin, processed routinely through graded alcohol and xylene series, and embedded in paraffin. Sections of 4 µm thickness were mounted on poly-L-lysine-coated slides and stained with hematoxylin-eosin. Neurons in the cornu ammonis CA1 and CA3 regions were evaluated semiquantitatively for pyknotic and degenerative changes and scored as absent (−), mild (+), moderate (++), or severe (+++).

For immunohistochemical evaluation, 4-µm paraffin sections mounted on poly-L-lysine-coated slides were deparaffinized in xylene and rehydrated through graded alcohol series. After washing with PBS, endogenous peroxidase activity was blocked with 3% H_2_O_2_ for 10 min. Antigen retrieval was performed in antigen retrieval solution at 500 W for 2 × 5 min. Sections were then incubated overnight at +4 °C with a primary anti-NOX1 antibody (Affinity Biosciences, Liyang, Jiangsu, China; Cat. No. DF8684) at a dilution of 1:250. As the secondary detection system, Large Volume Detection System: anti-Polyvalent, HRP (Thermo Fisher Scientific, Fremont, CA, USA; Cat. No. TP-125-HL) was applied according to the manufacturer’s instructions. Immunoreactivity was visualized using 3,3′-diaminobenzidine (DAB) as the chromogen. After counterstaining with Mayer’s hematoxylin, the sections were coverslipped with Entellan and examined under a light microscope. Immunopositivity in the cornu ammonis regions (CA1/CA2 and CA3) was scored semiquantitatively as absent (−), mild (+), moderate (++), or severe (+++). Histopathological scoring and semiquantitative immunohistochemical evaluation were performed by an experienced observer who was blinded to the experimental group allocation.

#### 4.3.10. Statistical Analysis

Statistical analyses were performed using IBM SPSS Statistics 22.0 and GraphPad Prism 6.0, as appropriate. Normality of continuous variables was assessed using the Shapiro–Wilk test. Normally distributed continuous data were analyzed using one-way analysis of variance (ANOVA), followed by Tukey’s post hoc test for multiple comparisons. Non-normally distributed continuous data were analyzed using the Kruskal–Wallis test followed by Dunn’s multiple comparisons test. Spike counts, first myoclonic jerk latency, generalized tonic–clonic seizure onset latency, and generalized tonic–clonic seizure duration were analyzed according to their distribution using either one-way ANOVA with Tukey’s post hoc test or the Kruskal–Wallis test with Dunn’s multiple comparisons test, as appropriate. Modified Racine scores and semiquantitative histopathological/immunohistochemical scores were treated as ordinal variables and analyzed using the Kruskal–Wallis test followed by Dunn’s multiple comparisons test. Histopathological and immunohistochemical scores were analyzed separately for CA1 and CA3 regions. Ordinal data are presented as median (IQR), whereas continuous data are presented as mean ± SD unless otherwise stated. A two-sided *p* value < 0.05 was considered statistically significant.

## 5. Conclusions

ML-171 attenuated PTZ-associated oxidant burden and apoptosis-related signaling in SH-SY5Y neuronal-like cells and in hippocampal tissue. However, it did not robustly improve the principal behavioral or electrophysiological seizure parameters in PTZ-kindled rats and therefore should not be interpreted as exerting a direct antiseizure effect under the present experimental conditions. The findings instead indicate that ML-171 primarily attenuates seizure-associated oxidative and apoptotic injury. The divergence among biochemical, histopathological, and NOX-1 immunoreactivity outcomes further indicates that these effects are endpoint- and dose-dependent and cannot be reduced to a simple linear model of NOX-1 suppression. Thus, the present data support modulation of selected injury-related pathways rather than suppression of seizure expression. Future studies incorporating direct NOX activity assays, NOX isoform profiling, ROS-specific measurements, pharmacokinetic analysis, sex-balanced animal designs, and time-course histological assessment are needed to define the precise mechanism and translational relevance of ML-171 in epilepsy-associated neuronal injury.

## Data Availability

The original contributions presented in this study are included in the article. Further inquiries can be directed to the corresponding author.
